# Exploring the differences between men’s and women’s perceptions of gender-based violence in rural Tajikistan: a qualitative study

**DOI:** 10.1186/s12905-021-01227-2

**Published:** 2021-03-04

**Authors:** Elizabeth A. Wood, Karina E. Wilson, K. D. Jacobs

**Affiliations:** 1 Department of Environmental and Global Health, College of Public Health and Health Professions, University of Florida, 1225 Center Drive, P.O. Box 100182, Gainesville, FL 32610-0182 USA; 2 College of Public Health and Health Professions, University of Florida, 1225 Center Drive, P.O. Box 100182, Gainesville, FL 32610-0182 USA; 3 Social and Behavioral Science, College of Public Health and Health Professions, University of Florida, 1225 Center Drive, P.O. Box 100182, Gainesville, FL 32610-0182 USA

**Keywords:** Tajikistan, Violence, Empowerment, Gender, Community empowerment drawing

## Abstract

**Background:**

Most women living in rural provinces of Tajikistan, specifically Khatlon, experience little to no opportunities for education and economic growth, making them vulnerable to gender-based violence (GBV). Unfortunately due to social norms that are bolstered by a patriarchal society, GBV has become tolerated and even normalized in rural areas. This study looks to investigate the differences in perceptions of violence as it relates to empowerment among men and women in rural Tajikistan.

**Methods:**

Data collection was done through participatory workshops and semi-structured interviews (SSI) that were led by Extension Home Economists, which lectured on gender, violence, and empowerment. Community Empowerment Drawings is a novel tool that has been used to further gather sensitive information that was not previously discussed. During this process, participant groups were asked to draw their perception of empowered versus disempowered women, later explaining the different characteristics and traits of both. Random participants across both genders were later contacted for a SSI to triangulate the data from the participatory workshops. This qualitative study implemented qualitative content analysis to explore the data inductively. Analysis of the drawings and transcripts from the workshops and SSIs included two researchers coding through an iterative process. Themes were stratified by men’s and women’s perceptions and codebooks were compared to ensure consensus.

**Results:**

Men and women from 12 villages participated in the Community Empowerment Drawings within each workshop, with 234 participants total. Results were stratified into two categories which were later broken down into notable themes: education, employment, decision-making, marital status, relationship wellness and respect, violence, mental health, and substance abuse. Major findings illustrated how disempowered women were perceived to have more exposure to men who experience alcohol abuse. This study found that differences in perceptions of empowerment between men and women remain—with men still holding onto the traditional power structure within a household and women challenging gender roles and mobility.

**Conclusion:**

Future studies may find engaging communities through drawings will yield more information regarding sensitive topics rather than traditional instruments. More support and advocacy are needed in areas of mental, neurological, and substance abuse disorders throughout rural Tajikistan.

## Background

After the collapse of the Soviet Union in 1991, many Central Asian countries faced economic insecurity [1]. Shortly after gaining independence from the Soviet Union, Tajikistan engaged in its own civil war from 1992 to 1997. This led to the downfall of the economy’s infrastructure and general welfare resulting in structural inequalities. Despite Tajikistan’s attempt to transition into a democratic government, its people suffered from financial instability and economic turmoil, which was exacerbated within rural regions [2]. Shortly after the civil war ended, a mass influx of migration to Russia of Tajik men occurred as they sought employment opportunities in Russia. Since Tajikistan is a developing, low-income country, a majority of its GDP is dependent on agriculture and farming [1]. Having this abrupt economic transition, gender roles began to shift in rural Tajikistan. As the migration of men increased, a growing number of women became responsible for Tajikistan’s agricultural sector without any adequate training. Without proper extension programs, Tajikistan began to face one of the highest malnutrition rates in Central Asia with nearly 75% of their population facing food insecurity [3]. With very little bargaining power, unemployment of men on the rise, and under education in rural areas of Tajikistan, women have often been relegated to low paid positions that are predominantly seasonal putting them in greater vulnerable positions [4]. Due to the social norms of the region, women working is often a sign of poverty and signals that the man of the household cannot provide sufficiently, leading to greater risk of gender-based violence (GBV) [1, 5].

The social role of gender is often enacted not only through the interactions between individuals of the same gender, but, more importantly, those of the opposite gender [6]. Gender roles in almost all cultures are complimentary to one another, often with strict boundaries and functions which define men as being the primary political and financial actors, while women are often restricted to domestic roles in order to serve as an aid to men [6]. Gender-based violence and intimate-partner violence (IPV) is a public health issue that includes emotional, physical, sexual, and financial abuse [7]. Violence against women is engrained in various societies around the world, exemplified through acts such as honor killings and female infanticide [7]. In Tajikistan, an alarming number of women believe that violence against women, specifically a wife, is warranted under certain conditions and is an integral part of marriage [8, 9]. These beliefs are perpetuated and reinforced by a patriarchal society and norms that limit a woman’s decision-making within the household [7]. Tajikistan borders Afghanistan and embodies fragments of Islamic radicalism, making it one of the more conservative Central Asian countries. Thus, most Tajik and Uzbek women lack autonomy and independence as a result of the heteronormative gender roles based on underlying patriarchal dominated religious roots [7]. Religious scriptures, texts, and societal norms are structured to enhance the patriarchy, portraying women as a subordinate possession [8]. Having this polarized power differentiation between men and women reinforces patrilineal structures within Tajikistan with violence against both younger and older women has becoming normalized and widely accepted [1, 7, 9]. In 2017 it was reported that 31% of ever married women have experienced some type of violence by their spouse, a 7% increase since 2012 [10]. However, this subject is largely understudied in Tajikistan where help-seeking behaviors are taboo and sexual transgressions bring dishonor [11].

As a result of male migration in rural regions of Tajikistan, many young women are forced to marry early to ensure they have a husband, which often leads to dropping out of school at an early age [12]. Due to the discontinuation of education and low socioeconomic status (SES), there is a strong proclivity for violence against women in the household [13]. Additionally, evidence suggests that low SES, financial instability, and little education, are risk factors for GBV and IPV [10, 13]. This is largely due to alcoholism in men who have low SES are more inclined to partake in GBV due to increased financial stress and lack of adequate or any psychiatric care [13]. Consequently, low-income Tajik males are more likely to face substance abuse issues and later resort to perpetrating IPV onto their female partners. As a result, women who reside in low-income areas are at a much higher risk of violence [9].

While there is existing evidence on the prevalence and determinants of GBV in Central Asian countries, there is limited research on the varying perceptions between men and women on violence [9]. Global organizations focused on the prevention of violence against women have begun to engage men as fundamental allies, marking a shift from men being seen predominantly as perpetrators to being considered valuable partners in the prevention efforts [14]. As men are significantly more likely to hold positions of power, influence, and authority, they have the potential to play a vital role in increasing awareness and shaping the discussion in a way that facilitates positive change in reducing violence against women [15]. In order to involve men more effectively, it is necessary to identify current perceptions, misconceptions, stereotypes, and attitudes that perpetuate GBV [16].

Collecting incidence and prevalence of GBV in rural Tajikistan not only brings risk to researchers and local liaisons, it is also unclear how reliable the data would be. Consequently, this study chose to focus on perceptions, which are shaped by social norms and knowledge, as a substitute for prevalence. Using the Social Ecological Framework, this study aims to investigate the differences in perceptions of violence as it relates to empowerment among men and women in rural Tajikistan. Specifically, what are the drivers of GBV as identified by men and women in Khatlon Province? Through investigating perceptions of GBV between Tajik men and women, researchers and in-country partners will be better equipped to design and implement targeted interventions that address societal gender norms.

### Reflexivity statement

This research, conducted by local Tajik partners and researchers within the University of Florida (UF), is important to be captured for this unique, understudied corner of the region. The lead author, E.W., has worked in this community over several years and has formed deep relationships with those in the region working in both development and research settings. K.W. and K.J. have worked closely with E.W. around issues of violence and women’s empowerment within this population. All three authors have been born and raised in the southern United States, are of upper-middle class, and white women. It is through the feminist principle that each researcher has the dual objective of sharing new knowledge and contributing to improved livelihoods within the region studied.

## Methods

### Research design

In May 2018, researchers from the University of Florida (UF) partnered with the United States Agency for International Development (USAID) Tajikistan Agriculture and Water Activity (TAWA) project to conduct this study in Khatlon Province, where the proportion of GBV is significantly the highest in the country [10]. The UF Institutional Review Board reviewed and approved this study prior to data collection and recruitment (IRB201800621). Data collection began in April 2018 through July 2018. This study used a phenomenology approach due to the lack of current literature that exists among this population specifically as a result of the intersection of gender hegemonies, social systems, and complex relationships with surrounding countries (e.g. Russia) [17]. UF researchers have worked previously with TAWA and the priority population within Khatlon Province, along the southern border of Tajikistan. There were 14 TAWA field agents, known as Extension Home Economists (EHEs), were trained by UF researchers in a train-the-trainer workshop format for one week prior to data collection. Due to working primarily with women in the villages, as well as the transience of male outmigration and gender norms, the EHEs are all women. However, they are viewed as trusted, local liaisons into the communities as a whole. The EHEs contributed to the project design, logistical support such as gathering the community members, and arranging times and locations to meet. They also acted as facilitators during this project and were primary data collectors. During each participatory workshop there would be one person facilitating and at least one other EHE taking notes and transcribing. Due to the various languages spoken throughout the villages (Russian, Tajik, and/or Uzbek) EHEs that could accommodate the specific language were assigned. Given their long relationship with those in the villages, the EHEs were aware prior to data collection who should be assigned to which village.

Prior to data collection, UF researchers trained EHEs in sensitivity, ethics, and how to prevent bias within research. Additionally, subject specific areas such as empowerment, gender, and violence were also discussed in detail with corresponding breakout activities. EHEs were consulted about appropriate language and translations prior to data collection. Since there is no direct translation for the word “empowerment” within Russian, Tajik, or Uzbek, words like “power” and “agency” (measures of empowerment) were used together to describe the subject. Additionally, since all of the EHEs are women there was a discussion around adding male administrators to data collection within the male groups specifically. However, this was immediately dismissed by TAWA partners as unknown men are considered much more of a threat within these villages than the known female Extension Agents. During the last day of training, the EHEs performed a mock participatory workshop that they would later carry out in the villages. Through this training, adjustments to the participatory workshop were made to ensure the EHEs were comfortable facilitating such a sensitive topic as well as to accommodate cultural differences. Following the training, the EHEs conducted participatory workshops in 12 villages throughout Khatlon Province through convenience sampling. The EHEs were tasked with selecting the villages as a result of having a much more comprehensive understanding of the sociopolitical climate in the area, which may impact safety.

### Participants

Men and women from each of the 12 villages were chosen to participate in the workshops separately, totaling 24 workshops. The region, Khatlon Province, was chosen as it had previously been established as a USAID’s Feed the Future Zone of Influence. The EHE’s have established relationships within each community and were able to act as trusted liaisons when recruiting participants. Prior to arrival, EHE’s would spread through word of mouth that they could be involved in a participatory workshop. From this point, men and women were chosen based on their willingness to participate in an hour long workshop, must be at least 18 years or older, and were residents of the village at the time of the workshop. Participants’ age were distributed evenly between 18 and 46 + with 44.4% identifying as men and 55.6% women, with the size of each participatory workshop ranging from 7 to 14 people. Men and women were segregated in order to accommodate traditional gender norms in the region.

### Data collection

The participatory workshops began with reading a script regarding consent to participate, allowing anyone to leave at any time without consequence. All participants were asked that if they would like to be re-contacted for an interview, they could leave contact information with the EHEs. The EHEs introduced themselves to anyone they were not familiar with and offered tea to create a more relaxed, informal environment. This was followed by a brief lecture that reviewed empowerment, gender, and defining violence (including gender-based violence). Once the lecture was completed, a cartoon Tajik-created public service announcement was shown to demonstrate how violence impacts everyone in the home. This prompted a discussion with participants around violence and whether it occurred within each community and in what form. Within this discussion participants were asked to draw on a large piece of paper split in half to show how they viewed an empowered woman compared to a disempowered woman. This technique, known as Community Empowerment Drawing (CED), is a strategy that was created to glean sensitive information from participants that may not have otherwise shared it. As part of the CED, participants must present their drawings as a group and explain why they drew certain traits and characteristics. Through this process, participants were able to draw and then describe empowerment and how it relates to violence. The EHE facilitators also probed, in a conversational style, with questions around violence specifically (see Additional file 1, Additional file 2, Additional file 3).

Participants were finally asked to rank the top three characteristics for both the empowered woman and disempowered woman in order to establish what the perceived priorities were. Each workshop was recorded, transcribed, translated from the language in which it was transcribed in (Russian, Tajik, or Uzbek) into English, and all CED papers were collected by researchers.

Following the participatory workshops, random participants who provided contact information to the EHEs were re-contacted at a later date, with permission, for semi-structured interviews (SSIs) to better understand the major themes captured through the CED as well as to triangulate the findings. EHEs contacted participants from the workshops using the methods preferred by the participant (e.g. word of mouth, phone, email). Of the participants who were contacted, nine were interviewed, four men and five women, about gender, empowerment, and violence as it related to the community. Questions were designed to be open-ended and semi-structured to allow for participants to expand and explain their answers thoughtfully. All interviews were conducted in-person and spoken in the participant’s native language and transcribed in real-time by a second EHE.

### Data analysis

Qualitative content analysis [18] was used by UF researchers to determine dominant themes throughout the 24 participatory workshop and 9 SSI transcripts. Through this iterative method, two researchers analyzed and coded transcripts independently to later stratify men’s and women’s perceptions of empowered and disempowered women. While there is a dearth of information around women’s empowerment within low-middle income countries, the decisional was made within the coding framework to have a binary approach due to language barriers within the Tajik, Uzbek, and Russian languages. As previously stated, since there is no direct translation from “empowerment”, constructs of empowerment were used within data collection. Following individual analysis, codebooks were compared between reviewers for inter-rater reliability and consensus. The confluence of the two sets of codebooks from the two reviewers resulted in the final themes presented in Tables 1 and 2. A comparison of perceptions between men and women was done primarily to establish key similarities and differences to how to approach violence and empowerment interventions in the future. The Community Empowerment Drawings (CEDs) were both used as a tool to allow participants to feel more comfortable about expressing their views and beliefs, as well as to verify what participants had stated verbally. However, no formal analysis was done with the CEDs.

**Table 1:** Demographic characteristics of the participatory workshops across villages

	Districts												
	Jomi	Dusti	Jalol Balkhi	Jaihun	Khuroson	Kushoniyon	Levakant (Guliston)	Levakant (Mehroobod)	Sharitruz	Qabodiyon	Nosiro Khusruv	Yovon	Total N (%)
*Age*													
18–25	7	7	3	7	3	1	9	7	4	1	2	–	51 (21.8%)
26–35	3	7	4	3	4	7	5	8	4	9	2	1	57 (24.4%)
36–45	4	5	6	2	3	2	2	1	6	5	9	10	55 (23.5%)
46 +	4	5	9	9	9	5	4	1	5	3	8	9	71 (30.3%)
*Sex*													
Male	9	10	10	8	10	7	–	17	7	6	10	12	106 (45.3%)
Female	9	14	12	13	9	8	19	–	13	12	11	8	128 (54.7%)
*Occupation*													
Farmer	14	22	22	20	17	13	19	3	18	18	10	16	192 (82.1%)
Housewife	–	2	–	–	–	2	–	–	–	–	11	1	18 (7.7%)
Student	–	–	–	–	–	–	–	6	–	–	–		6 (< 1%)
Teacher	2	–	–	–	–	–	–	7	2	–	–	1	10 (< 1%)
Cook	1	–	–	–	1	–	–	–	–	–	–	–	2 (< 1%)
Local government	1	-	-	1	1	-	-	1	-	-	-	1	5 (< 1%)
Not reported	-	-	-	-	-	-	1	-	-	-	-	-	1 (< 1%)

**Table 2:** Male versus female perceptions of empowered women

Theme	Qualitative content analysis		
	Male group perception	Female group perception	Outcome COMPARISON
Education	An empowered woman is educated with a university degree “She was grown up in a very good family, has diploma, her relatives have high education”	An empowered woman is educated with a university degree “She is smart and educated. She has received a high education and earned a university degree”	Both males and females have similar perceptions of empowered women having higher education, specifically graduating with a college degree. Empowered women have completed their primary and secondary education. A diploma was very symbolic of empowerment
Employment	Empowered women have a permanent and stable job that offers a sufficient income. It is common for them to own a business, such as a factory or food shop. These women also own their land and draw profits from it “She has a permanent job and owns a factory. She also has income from her livestock and orchard.” “Her husband is a businessperson.” “[An empowered woman] has her own food shop, orchard and car. She is a businesswoman. She controls income from the shop and her husband shares his income with her for the household and children.”	Empowered women have a permanent and stable job that offers a sufficient income. It is common for them to own a business, such as a factory, fashion line, bank, or medical practice “She is designer, fashion designer… [She] has medical degree and is a doctor… [She] works in a bank… [She] is a chairwoman.” “She has permanent job, big house, and truck. An empowered woman does not have any difficulties as she has a husband, job, children, and delicious food. She can buy whatever she wants.”	Both males and females have the perception of empowered women having their own stable income and a permanent job. However, the female group specified that empowered women can work in many diverse environments such as finance, medicine, fashion, and politics. The male group only mentioned women working in factories or owning land. Male groups varied on what they viewed as the women’s role. While some groups shared that women shared and/or controlled the income with their husbands, other groups specifically mentioned that only their husbands worked—and that is what made them empowered
Decision-making	Empowered women do not make their own independent decisions and their husbands do not solely control the income. Instead, they make joint decisions with their husbands. These women have a mutual understanding within their marriage and consult with their partner when it comes to financial decisions. The husband and wife have control over their respective income, however, they both discuss their family issues and make decisions together “The budget is controlled by both wife and husband, as they have a mutual understanding of finances in their relationship… when it comes to making financial decisions, both husband and wife will consult with one another.”	Empowered women can make joint decisions with their spouse, but it is not commonly perceived. Instead, empowered women are more likely to make their own independent decisions. This is executed by managing the household by herself and controlling the entire household budget. There was also one mention of an empowered women whose budget is solely controlled by her husband “… [She] can buy whatever she wants because she manages her household herself… [She] has her own income so she controls it by herself.”	There was no general consensus on decision-making amongst the groups. The male group perceive that empowered women engage in joint decision-making with their husbands. This differs with the predominant female perception, as they emphasized the idea of independent decision-making. The female group stated that if an empowered woman has her own income, she is able to manage that money herself and could spend her earnings however she wanted. There was no mention of joint decision-making in the female group, however, there was one mention of the husband independently controlling their budget
Marital status	Empowered women are married and have a husband	Empowered women are married and have a husband	There is a consensus between both groups that empowered women are married and do not engage in divorce
Relationship wellness and respect	Empowered women respect their husband and treat them well. There is no mention of this being reciprocated by the husband “An empowered woman firstly respects her husband. His wife and children respect him.”	Empowered women have a mutual respect system with their husband. Both husband and wife share love and respect for one another “Both, wife and husband, respect each other.” “Wife and husband have mutual understanding in the family.”	The male group perceives empowered women to have a one-sided respect system where they exclusively respect their husband, but fail to state whether women receive anything in return. This differs from the female group where empowered women have a mutual respect system within their marriage
Violence	It is common for empowered women to have experience with violence during their childhood, but do not face it later in life. It is perceived that it is less likely for empowered women to experience violence because they have a family with a stable income. However, most empowered women have faced violence at some point in their lives, typically during their childhood “Currently, she does not experience violence in the family. She only had conflict with other kids in childhood. Her children do not experience any types of violence… [She] has a friendly family that respects each other and her family has enough income.”	Empowered women are perceived to rarely experience violence during their childhood. If childhood abuse occurred, it was perpetrated by their mother. It is commonly perceived that empowered women do not experience violence throughout their lives because the family has respect for one another “She did not experience any types of violence in the past and does not experience now. [Her] family respects her and her husband loves her very much.”	The male group perceives empowered women to have experience with violence, typically during their childhood. However, the female group perceives empowered women to be free of violence throughout their lives
Mental health	Empowered women are happy and have a good life. Their mental health is in a good state “Her life is very good… [She] is happy and has good health.”	Empowered women are happy and have a good life. She will have positive thoughts and ideas on life. Their mental health is in a good state “She is happy… [She] has very positive thoughts and ideas for perspective life.”	There is a consensus between the male and female groups that an empowered woman’s mental health is in a good state. Therefore, leading to positive thoughts and to a “good” life

## Results

Men and women across 12 villages participated in the Community Empowerment Drawings within each workshop, with 234 participants total. There was an even distribution of men and women that participated in these workshops, with a slightly higher attendance from women over 46 years of age as indicated in Table 1. The nine semi-structured interviews were used to triangulate the workshops, however, no quotes will be shared from these interviews specifically. As previously discussed, the participatory workshops, which include the Community Empowerment Drawings, form the underpinnings of this study. Upon analyzing the transcripts and CED, results were stratified into two categories: perceptions of empowered women and perceptions of disempowered women (as viewed by men and women separately) and broken down further into salient themes. Major themes manifested from Men’s versus Women’s Perceptions of Empowered Women (Table 2) included education, employment, decision-making, marital status, relationship wellness and respect, violence, and mental health. Themes that were included in Men’s versus Women’s Perceptions of Disempowered Women (Table 3) were education, employment, decision-making, marital status, relationship wellness, violence, mental health, and substance abuse. While there is substantial overlap in themes between the two categories, there are significant differences, as illustrated in Tables 2 and 3.

**Table 3:** Male versus female perceptions of disempowered women

Themes	Qualitative content analysis		
	Male group perception	Female group perception	Outcome comparison
Education	It is perceived that disempowered women have limited to no education, which causes her to lack life skills. It is also common for a disempowered woman to only have her primary or secondary education as a result of dropping out “She has no education… [She] does not have any skills… [She] has only primary education… [She] has secondary education but could not continue her education.”	It is perceived that disempowered women have no education at all, which causes her to lack basic life skills. They were never able to pursue an education because they never attended school “She has no education at all… [She] does not have any skills… [Her] husband and family have no education.”	The male group perceives disempowered women to have some experience with school, they just did not receive their diploma at the college or university level. It was commonly perceived that disempowered women went to primary school, but did not complete it. This differs from the female group because they perceive disempowered women as completely uneducated, and did not attend school at all. Despite these differences, both groups agreed that many disempowered women do not hold diplomas
Employment	Disempowered women are perceived as unemployed and do not own property. There are some instances where if the woman is married, her husband has migrated for temporary work in Russia. If the husband has seasonal work, it is common for him to not send money to his family. Disempowered women who have temporary employment or a seasonal job work in agriculture. If the woman has children, she will resort to pulling the children from school to assist with field work. Her children will typically work in the field their entire childhood and oftentimes never receive an education “She does not have a job, [her] husband has temporary work” “[Her] husband is working on the tractor seasonally and sometimes as a taxi driver, and he migrates to Russia in the winter.” “Her husband is on migration and does not send any money to support them.” “Her children work in the field since childhood to earn money so that they can support their mother… [She] has a seasonal job of working in the neighbors’ lands to earn money and in her kitchen garden, where she plants some vegetables.”	Disempowered women are perceived as unemployed and do not own property. It is common for them to have a husband that is unemployed and lethargic because he does not wish to work. Unemployed husbands do not help their wives with household work, as she is expected to complete it by herself. If the husband has temporary employment through migration, he does not send money home to support his family. If the disempowered woman has temporary employment herself, it is typically seasonal work in her kitchen garden, in the field, or on her neighbor’s land to earn money and food. The hours range from the early morning until the late evening, and the salary is just enough for daily expenses. If she needs help on her own field, she resorts to child labor and forces her children to work “Her husband has temporary work.” “She does not have a permanent job. She has seasonal work in the kitchen garden. She is working on a cotton field and doing heavy work. Her husband is very lazy because he does not want to work.” “Her children work in the field since childhood to help… [She] forces them to work.”	Both male and female groups had a general consensus that disempowered women are often unemployed. If she has temporary work in her own kitchen garden or on her neighbor’s land, she orders her children to work with her. Both groups also agreed that if the husband is on seasonal outmigration, he would not send any money to support his family. The only difference amongst the male and female groups is that the female group mentioned instances where the husband was unemployed because he is lazy and apathetic. The male groups never discuss husbands being unemployed, only having temporary employment
Decision-making	Disempowered women have no voice when it comes to decision-making. Their income is controlled independently by their husband “Her income is controlled by her husband.”	Disempowered women have no voice when it comes to decision-making. Their income is controlled independently by their husband. If her husband is on migration, then she will manage the household by herself and make financial decisions independent of him “Her husband controls the seasonal income.” “Her husband is on migration so she controls the income.”	Both groups agreed that the disempowered woman’s husband will have control over their income and make the decisions himself. However, the male group never mentioned an instance where the disempowered women make decisions independent of their husband when he is on migration. The female group explained that disempowered women are able to make their own independent decisions when their husband is not present
Marital status	Disempowered women are divorced, widowed, or married to a husband on migration or unemployed “She is widowed.” “She is married but her husband is on migration” “[She] is divorced.”	Disempowered women have a husband, but he is unemployed or on migration. There are some instances where she is a widow	Both groups agreed that disempowered women are widowed, have a husband who is unemployed, or a husband on migration. The main difference between groups bring the male participants noted that disempowered women may also be divorced
Relationship wellness	Disempowered women do not respect their husbands “She does not respect her husband.”	Disempowered women do not respect their husbands because she is afraid of him “Her husband is an alcoholic. She does not respect him… [She] is afraid of her husband.”	Both male and female groups agree that disempowered women do not respect their husbands. However, the female group goes in depth about why this is. Disempowered women do not respect their husband because they are fearful of him, thus amplifying negative attitudes towards him
Violence	Disempowered women have had experiences with violence during their childhood such as psychological, physical, and financial abuse within their family. It is also common for them to face domestic violence within their marriages as a result of their husbands engaging in continued substance abuse (typically alcohol). This abuse is then adopted by the woman and perpetrated against her children “Her husband is an alcoholic and beats her every day because she is lazy and does not do anything in the house.” “Her family was poor and her father was an alcoholic so he did not take care of his family. Now she and her family experience psychological and financial violence.”	Disempowered women face domestic violence and psychological, physical, and financial abuse almost daily. It is common for her husband to beat her and their children. The children also face the same kinds of abuse and are forced to work in the field with their mother “She experiences psychological, physical and financial violence almost every day. Her husband beats her every day. Her children also experience psychological, physical and financial violence as they are forced to work in the field. Their father beats them as well.”	Both male and female groups agreed that disempowered women experience domestic violence and are physically abused by their husbands. They have also experienced psychological and financial abuse throughout their lives. However, the male group specifies that the mothers beat their children whereas the female group state that the husband is the perpetrator. There is a difference in perception between genders on child abuse and its perpetrators
Mental health	Disempowered women do not have a stable mental health. They are usually stressed and upset “She had a troubled childhood.” “She is always stressed and depressed, nervous.”	Disempowered women do not have stable mental health. It is common for them to be angry and stressed “She is very nervous, angry, and always stressed… life is hard for her.” “She is suffering.”	There is a consensus from both male and female groups that disempowered women do not have stable mental health and are constantly feeling stressed
Substance abuse	It is perceived that disempowered women first experience instances of substance abuse during their childhood, where their father is an alcoholic. Disempowered women also face substance abuse in their marriage, specifically with their husbands. The husband typically faces issues with alcoholism “Her husband is alcoholic… [Her] father was alcoholic.”	Disempowered women also face substance abuse in their marriage, specifically with their husbands. The husband typically faces issues with alcoholism which results in lack of income for the family “Her husband is an alcoholic… [Her] husband does not help her and he spends his money on alcohol.”	Both groups agree that disempowered women face substance abuse issues with their husbands. However, the male group specifically mentioned that disempowered women have experiences with substance abuse during childhood with their fathers as well

### Empowered women

#### Education

There was a consensus between both men and women that empowered women will have accomplished obtained a level of higher education, specifically a college level degree. As one male stated, “She was grown up in a very good family, has diploma, her relatives have high education.” Participants across all groups for both men and women drew and discussed having a diploma so exceedingly often, that it was deemed to be especially symbolic of an empowered woman. For example, one female participant exclaimed, “She is smart and educated. She has received a high education and earned a university degree.”

#### Employment

Both participant groups agreed that empowered women have a stable occupational status that is full time, rather than seasonal or temporary. Empowered women never have to work a temporary job, as it was implied that empowered women have careers with a steady income. As one male participant noted, “She has a permanent job and owns a factory. She also has income from her livestock and orchard.” One notable difference between participant groups is that the women’s groups emphasized how empowered women are employed in diverse areas of work, with potential jobs ranging from finance to fashion to medicine, among others, as recounted by a female participant by stating, “She is designer, fashion designer… [She] has medical degree and is a doctor… [She] works in a bank… [She] is a chairwoman.” However, the men’s groups only discussed empowered women working in factories or with the land and did not include other career paths.

#### Decision-making

Perceptions of decision-making among empowered women varied by participant group. The men’s groups believed that empowered women make joint decisions with their husbands as one man stated here: “The budget is controlled by both wife and husband, as they have a mutual understanding of finances in their relationship… when it comes to making financial decisions, both husband and wife will consult with one another.” In cases like this, husband and wife have their own respective income, yet they decide as a couple to make financial decisions for the household. This contrasts with the women’s groups, where there was a general consensus that empowered women make their own independent decisions regardless of their husband’s input (with their own generated income specifically). It was emphasized that if a woman has her own income, she should have the ability to spend it however she desires. According to one of the female participants, “… [She] can buy whatever she wants because she manages her household herself… [She] has her own income so she controls it by herself.” There was one outlier that stated that an empowered woman still relies on the husband to make independent decisions. Overall, there was no mention of joint decision-making amongst the women’s participatory groups.

#### Marital status

There is agreement across all groups that an empowered woman is married and has a family. While this study did not capture self-reported religious ideology, Tajik facilitators had identified certain villages as more religiously conservative than others. In the context of this study, religious ideology refers to the degree in which an individual practices their religion on a daily basis. For example, the number of children varied within groups, with more religiously conservative groups suggesting anywhere from four to six children, and less religiously conservative groups stated two children. All participants agreed empowered women do not experience divorce, nor are they widows.

#### Relationship wellness and respect

There was no general consensus amongst the participatory groups when it came to the reciprocity of respect within intimate partner relationships. The men’s groups held a single-sided respect system viewpoint, where they emphasized the importance of empowered women respecting their husband. Within this viewpoint, there was no mention of a husband respecting his wife as one man states, “An empowered woman firstly respects her husband. His wife and children respect him.” This differs from the women’s groups majority perception that a mutual-respect system exists in an empowered woman’s relationship, where both partners are given respect as indicated by one female participant, “Both, wife and husband, respect each other.”

#### Violence

The men’s groups highlighted that despite a woman being empowered, she still experienced violence during childhood, which includes bullying from peers and household abuse. One male explains “Currently, she does not experience violence in the family. She only had conflict with other kids in childhood. Her children do not experience any types of violence… [She] has a friendly family that respects each other and her family has enough income.” This perception contrasts with the women participants, where they never mentioned a case where an empowered woman faces violence. Within the women’s groups, a sign of empowerment is to be without violence, as stated here “She did not experience any types of violence in the past and does not experience now. [Her] family respects her and her husband loves her very much.” Women who are empowered do not experience violence throughout their lives and will translate this stable environment to their children.

#### Mental health

While mental health was only subtly and briefly spoken about, researchers thought it important to include. Both participatory groups agreed that empowered women have stable mental health. There was a consensus that a sign of empowerment is to be happy, thus creating constant positive thoughts. It is perceived that having good mental health will amplify these positive thoughts, leading to a better quality of life emotionally. When asked to elaborate, a better quality of life often included how many friends an individual has, how happy they are in public, if they attend parties held by friends and family.

### Disempowered women

#### Education

There was a general consensus amongst the male and female participatory groups that a disempowered woman has never attended school, therefore she has little to no basic life skills. However, there were some reports from men’s groups that explained how disempowered women may have been enrolled in primary education, but never received a diploma, making them under educated, as state here “She has no education… [She] does not have any skills… [She] has only primary education… [She] has secondary education but could not continue her education.” Whereas, the women’s participatory group agreed that disempowered women receive no education. One female participant explained, “She has no education at all… [She] does not have any skills… [Her] husband and family have no education.”

#### Employment

Both men’s and women’s groups agreed that a disempowered women is often unemployed. If a woman did work, she would farm in her small kitchen garden or work on her neighbor’s land for income and it would be seasonal, not permanent. One male participant stated “[Her] husband is working on the tractor seasonally and sometimes as a taxi driver, and he migrates to Russia in the winter.” The participants also agreed that if a disempowered woman had a husband who had temporary work through outmigration, he would not send his family remittances from Russia. One notable difference between both groups is that the female participants discussed how disempowered women have husbands who were unemployed and had no will to work because they were lethargic. A female participant explained “She does not have a permanent job. She has seasonal work in the kitchen garden. She is working on a cotton field and doing heavy work. Her husband is very lazy because he does not want to work.” There was no mention of husbands being unemployed from the male participants, as it is perceived that they at least have a temporary job.

#### Decision-making

When it comes to decision making, it was agreed that a disempowered woman’s husband will have control over their income. It is typical for a disempowered women to have little to no control over the financial decisions and for the husband to independently make decisions himself. One major difference between the groups is that the female participants shared that a disempowered woman is able to make independent decisions, only if her husband is on migration.

#### Marital status

The two participatory groups agreed that disempowered women are more likely to be widowed, married to a husband who is unemployed, or has a husband who is on migration in Russia. Despite these similarities, the male participants mentioned that disempowered women is divorced. The female group did not mention divorce as a precursor for the disempowerment of women.

#### Relationship wellness and respect

There was a general understanding that disempowered women do not have healthy relationships with their husbands. Both men’s and women’s groups emphasized that disempowered women do not have respect toward their husbands, largely due to substance abuse or unemployment as indicated by one male participant who added “She does not respect her husband.” The only discernible difference amongst the groups was that the female participants explained that wives are often fearful of their husbands, which minimizes the amount of respect that they have for him.

#### Violence

There was an agreement between both groups that a sign of disempowerment is domestic abuse and experiencing various types of violence from a husband. Specifically, disempowered women experience psychological, physical (sexual), and financial violence throughout their marriage and youth, having major implications on their livelihood. However, one particular deviation between the groups is that the male participants perceive disempowered women not only as a victim, but as a perpetrator. It was repeated across different men’s groups that disempowered women beat their children. Although women’s groups explicitly state that the husband is the one that physically abuses the children. There is a clear distinction between men’s and women’s perceptions on the gender of the perpetrators and child abuse.

#### Mental health

Both participant groups agreed that disempowered women have mental health issues. It was made clear that a sign of disempowerment is to constantly feel apprehensive, indignant, and discontent toward life. A disempowered woman has no friends, is disconnected from family, and does not have a strong social network according to responses. One male respondent indicated “she had a troubled childhood.” While a female respondent states “[a disempowered woman] is suffering.”

#### Substance abuse

All participant groups acknowledged that disempowered women are exposed to substance abuse through their husbands. A male participant shared, “Her husband is alcoholic… [Her] father was alcoholic.” However, only the male participants noted that disempowered women are exposed earlier in life through their fathers.

## Discussion

### Career outlook and decision-making among women

The purpose of this study was to explore the contrasts in the perception of GBV between men and women, as it relates to empowerment. The women in this study define empowerment as having an autonomous choice on what career path they choose, not just the acquisition of a job [19]. This could potentially lead to more women, especially in rural areas, challenging patriarchal societal norms wherein women may only obtain a fixed amount of occupational success. Despite women wanting diversified career paths, men emphasized that an empowered woman has full-time employment in a formal sector where salaried labor is guaranteed and the workers are protected under legislation [19]. For example, factory work was brought up in discussions, however, it is also considered a low-paid, male-dominated occupation. The women who employed in formal sectors are oftentimes highly educated and skilled, yet they earn lesser salary than a man with minimal achievements due to gender-occupational segregation [19].

The agricultural sector provides employment for 66% of Tajikistan’s working population, with women now having the triple burden of working within agriculture, tending to the household, and fulfilling the roles of motherhood [1]. As a result it is fairly common for disempowered women to have temporary or seasonal work in nearby gardens or fields, utilizing her children as a primary source of labor. This fragmentation of the family magnifies the gender stratification in Tajikistan, as the male of the household fulfills his breadwinner role and the female is responsible for the home and children despite also working. Although the agreement among groups that disempowered women are unemployed or work in agriculture, the women’s participatory groups mention instances of men who are unemployed and act as the major driver of the women's disempowerment.

The women in this study were less likely than men to want joint decision-making within the household regarding finances. Tables 2 and 3 illustrate how despite women wanting diversified career choices, they were less likely than men to want joint decision-making within the household regarding financial decisions. While the underpinnings of women’s empowerment has been laid with increasing autonomy and agency, joint decision-making has proven successful at increasing mutual respect in the household [20]. Egalitarian decision-making has also been shown to be an effective approach to intra-household decision-making among dual-headed households [21]. This female perspective may have been pervasive due to another differential between the groups—respect and how it should be shared. Male groups were more likely to state that respect for a husband by his wife is mandatory, regardless of she is empowered or disempowered; however, the male groups neglected to describe whether women (disempowered or not) should also receive respect. This appears to be contradictory in that male groups predominantly agreed mutual decision-making was most beneficial in financial situations, while also glossing over whether females should receive respect within in the household. This perspective suggests that men, at least in the household, remain having power over their wives with women in their traditional marginalized position. The unequal power distributions in the household can limit access to education, economic improvements (e.g. employment for women), and other resources; all of which are needed for increased empowerment [20, 21].

### Substance abuse and mental health

Men’s and women’s participatory groups had a general consensus that disempowered women, exclusively, face exposures with substance abuse in their relationships with men, notably with alcohol. Despite Tajikistan being a predominantly Muslim country, alcohol is still bought and sold openly, although it is still considered culturally taboo for a woman to be seen consuming alcohol. Post-soviet countries, such as Tajikistan, rely on the prospering economy in Russia for various work opportunities, where high drinking frequencies often leads to intoxication [21]. Therefore, with over 40% of the Tajik population (largely men from rural areas) going to Russia for employment, they become exposed to Russian culture and norms [1, 6, 22]. As a result, they are more prone to engage in risky behaviors due to their encounters of prejudice in Russia, lack of familial support paired with social isolation, and economic instability [23, 24]. Despite encountering instances of xenophobia and nationalist violence, it has become common for migrants to neglect their mental health so that they can prioritize economic engagements. Empirical studies have observed that substance abuse disorders typically occur in Muslim migrants who reside in Russia [23, 24]. Further evidence shows that 96.2% of the population do not receive care for their substance abuse disorders, with it typically taking an average of 13 years to seek treatment [25]. These individuals are married, have received education from secondary school or less, and identify as economically disadvantaged. Nearly 61% of these men are of working age (18–49), have living situations in their home country where they have just enough money for food and basic necessities [25]. This drinking culture is later adopted by Tajik men, where they bring home these behaviors to their families when or if they return.

In many Central Asian countries, mental health literacy is minimal and negative images of mental illness are common in the media. Consequently, stigmas exist and are sometimes bolstered by cultural or traditional beliefs. Even while abroad, access to mental health services or health services in general, for labor migrants has been well documented as limited to non-existent compared to non-migrants within the same country [23, 26–35]. Additionally, gender differences in mental health literacy emphasizes a wide gender gap between male and female mental health. For example, older males have a lower mental health literacy, as they are unable to distinguish psychological distress from chronic mental health issues [25]. As a result, this further normalizes the culture of alcoholism and poor mental health in males, thus potentially manifesting into gender-based violence and abuse towards their female counterparts.

### Perpetrators of violence

Despite the normalization and even acceptance of GBV amongst women in Khatlon [9], it was reported across groups that disempowered women are constantly stressed, which could later manifest into anxiety and mood disorders. Furthermore, disparities between suicide rates in rural and industrialized provinces in Tajikistan cause women who reside in underdeveloped areas to have higher rates of suicide, as they have lower health literacy and limited access to health care services [12]. It is also common for Tajik women to become desensitized to violence since it has become habitual amongst other women in their lives, such as sisters, mothers, and/or aunts [12]. Victim-blaming is also a common practice among men and women, where even medical practices and social support services may refuse to offer assistance to battered wives [12]. Violence against women, of any type, perpetuated by the husband is often perceived as “justifiable” by women in Tajikistan, with older women often blaming young mothers specifically [12]. Battered women rarely seek help as a consequence of feeling blamed of their victimization, consequently, suicide is often the only escape from abuse where women find power and control and can rebel against socialized gender norms [11, 12, 36]. However, it is important to disentangle what participants in this study perceive with reality. This study does not intend to imply that disempowered women experience GBV because they are disempowered, nor that those that have experienced GBV are disempowered women. This study’s objective is to share and compare perceptions of violence as viewed by men and women.

Another gender difference amongst the groups was the perception of how empowered women encounter violence in their lives. Men reported that despite a woman’s empowerment, it was common for her to have experienced abuse during her childhood with either her father or instances of bullying in school. This is significantly different from the way women perceive empowerment, as they mentioned that empowered women never face violence in their lives. In Tajikistan, daughters are expected to adhere to the needs and expectations of the male figures within their household, such as their fathers, brothers, and in-laws [12]. As a result, this behavior is a social norm with deep, traditional roots within rural communities making it rare for a woman from this background to have never experienced or had exposure to violence.

Due to the qualitative nature of this study, the small sample size does not allow for generalizability outside of the priority population. Other limitations to this study that should be considered include how the sample was collected using pre-established women’s groups that in-country partners had been working with and the potential for the interpretations of words to be lost in translation, despite best efforts to avoid this. The semi-structured interviews were specifically not recorded for identification purposes, therefore, transcripts from the interviews may not be as representative and accurate as the participatory workshops. Additionally, while it was considered during study design, researchers found it inappropriate to determine the nuances of religious ideology between villages due to potential bias. Therefore, while there may have been a variance between how each village practiced Islam (specifically how conservative) this information was not captured in this study. Finally, though these findings were shared with local partners and integrated into new interventions within the region, due to the analysis being performed by non-Tajik researchers, there is always the risk of misinterpretation due to implicit biases and lived-experiences of the researchers.

## Conclusion

This study builds upon previous research around women’s empowerment by including men and gathering their perceptions of empowerment, gender, violence, and decision-making. Recognizing the importance of the male perspective has been recommended by several studies in the past as men and women tend to have difference of opinions and perspectives in regards to women’s empowerment—as this study has shown [21, 37–39]. Future extension-related programs should conduct workshops or seminars with a culturally sensitive curriculum around the impact of financial stressors placed on unemployed men from the region. It is imperative to include men in discussions around reducing poverty, increasing educational and/or trade opportunities. With consideration to the historical and political climate of Tajikistan, understanding the patrilineal social system is essential in designing successful interventions.

With the global burden of disease from mental, neurological, and substance abuse disorders anticipated to increase from 12.3% in 2000 to 14.7% in 2020, more support and advocacy is needed to address the stigma within vulnerable and marginalized populations [38, 39]. The lack of advocacy from stakeholders, limited access to health care, crisis centers, and legal services in addition to the normalization of GBV in relationships, are the suspected causes of why many women do not seek out medical or professional help [9, 10, 12]. According to our findings, it would be beneficial to include men within more interventions that address substance abuse issues, mental health services, and job-creation opportunities. By including men, we are better equipped to recognize and address the distal factors the influence GBV within the household. The EHEs hold a unique role with the local communities as they are both respected and considered agents of change since they are all Tajik. Through their efforts, among other partner-led interventions, the nexus of gender norms and likelihood of violence within the home may decline.

## Supplementary Information

**Additional file 1.** Prompted questions.

**Additional file 2.** Checklist for facilitators conducting community workshops.

**Additional file 3.** Interview prompt for community workshops.

## Data Availability

The data generated and/or analyzed during this study are not publicly available due to restrictions related to the sensitive nature of the topic and limit risk for participants of the study.

## Abbreviations

CED: Community Empowerment Drawing
EHE: Extension Home Economist
GBV: Gender-based violence
HIV: Human immunodeficiency virus
IPV: Intimate Partner violence
SES: Socioeconomic status
SSI: Semi-structured Interview
TAWA: Tajikistan Agriculture and Water Activity
UF: University of Florida
USAID: United States Agency for International Development
