# Correction to: Exploring the differences between men’s and women’s perceptions of gender-based violence in rural Tajikistan: a qualitative study

**DOI:** 10.1186/s12905-021-01266-9

**Published:** 2021-03-24

**Authors:** Elizabeth A. Wood, Karina E. Wilson, K. D. Jacobs

**Affiliations:** 1 Department of Environmental and Global Health, College of Public Health and Health Professions, University of Florida, 1225 Center Drive, P.O. Box 100182, Gainesville, FL USA; 2 College of Public Health and Health Professions, University of Florida, 1225 Center Drive, P.O. Box 100182, Gainesville, FL USA; 3 Social and Behavioral Science, College of Public Health and Health Professions, University of Florida, 1225 Center Drive, P.O. Box 100182, Gainesville, FL USA

## Correction to: BMC Women’s Health (2021) 21:91 10.1186/s12905-021-01227-2

Following publication of the original article [1], we were notified that the Reflexivity statement needs to be revised.

Originally published statement: “All three authors have been born and raised in the southern United States, are of upper-middle class, and white women.”

Corrected statement: “All three authors have been born and raised in the Southern United States. K.W is an Asian-White biracial woman in lower-middle-class.”

The original article has been corrected.
