# Supplementary material for: Exploring the differences between men’s and women’s perceptions of gender-based violence in rural Tajikistan: a qualitative study
Source: BMC Womens Health. 2021 Mar 4;21:91. doi: 10.1186/s12905-021-01227-2 (PMC7934274; doi:10.1186/s12905-021-01227-2)
Supplement: Supplementary file 1 — Additional file 1. Prompted questions. [file 12905_2021_1227_MOESM1_ESM.docx]

Prompted Questions

1. What does the most empowered woman/ man look like?
2. What does his/ her family look like? Does he/ she have children? Is the spouse working abroad?
3. How does he/ she treat their spouse? How does he/ she treat their children?
4. What assets does he/ she have?
5. What was his/ her childhood like?
6. Does he/ she have lots of friends in the community? Does he/ she have lots of relatives in the community?
7. Has he/ she experienced violence?*
8. Has his/ her children experienced violence?*

*GBV-related questions

Russian:

Подсказанные вопросы

1. Как выглядит женщина / мужчина с наибольшими возможностями?

2. Как выглядит его / ее семья? Есть ли у него дети? Супруг работает за границей?

3. Как он / она обращается со своим супругом? Как он / она относится к своим детям?

4. Какие активы у него / нее есть?

5. Каким было его детство?

6. Много ли у него / нее друзей в сообществе? Много ли у него / нее родственников в общине?

7. Испытывал ли он / она насилие? *

8. Испытывали ли его / ее дети насилие? *

Tajik:

Саволҳои фаврӣ

1. Зан / марди аз ҳама ваколатдор ба чӣ монанд аст?

2. Оилаи ӯ ба чӣ монанд аст? Оё ӯ фарзанд дорад? Оё ҳамсар дар хориҷа кор мекунад?

3. Вай ба ҳамсари онҳо чӣ гуна муносибат мекунад? Вай ба фарзандони онҳо чӣ гуна муносибат мекунад?

4. Вай чӣ дороиҳо дорад?

5. Бачагии ӯ чӣ гуна буд?

6. Оё ӯ дар ҷомеа дӯстони зиёд дорад? Оё ӯ дар ҷомеа хешовандони зиёд дорад?

7. Оё ӯ зӯровариро аз сар гузаронидааст? *

8. Оё фарзандонаш ба хушунат дучор омадаанд? *

Uzbek:

Tezkor savollar

1. Eng vakolatli ayol / erkak qanday ko'rinishga ega?

2. Uning oilasi qanday ko'rinishga ega? Uning bolalari bormi? Turmush o'rtog'i chet elda ishlayaptimi?

3. U turmush o'rtog'iga qanday munosabatda bo'ladi? U bolalariga qanday munosabatda bo'ladi?

4. U qanday mol-mulkka ega?

5. Uning bolaligi qanday bo'lgan?

6. Uning jamiyatda ko'plab do'stlari bormi? Uning jamiyatda ko'plab qarindoshlari bormi?

7. U zo'ravonlikni boshdan kechirganmi? *

8. Farzandlari zo'ravonlikni boshdan kechirganmi? *
