# Supplementary material for: Exploring the differences between men’s and women’s perceptions of gender-based violence in rural Tajikistan: a qualitative study
Source: BMC Womens Health. 2021 Mar 4;21:91. doi: 10.1186/s12905-021-01227-2 (PMC7934274; doi:10.1186/s12905-021-01227-2)
Supplement: Supplementary file 2 — Additional file 2. Checklist for facilitators conducting community workshops. [file 12905_2021_1227_MOESM2_ESM.docx]

Interview questions

Length of interview: 60-75 minutes

1. Could you tell me about yourself?
2. How long have you lived in this community?

Empowerment-related:

1. Do you think the women in your community are empowered or disempowered? 3a. Could you explain why do you think so?

- *If the answer is that women in the community are empowered, please ask:*

1. Are there any women in the community that are disempowered? 4a. Could you explain why do you think so?
2. In your opinion do you think a disempowered woman is a victim of violence? 5a. Can you explain why?
3. Could you tell me how a disempowered woman can become empowered in your village/district?
4. Do women make/participate in decision-making within households in your community?

*For additional probing:*

- *If yes:* Can you describe the type of decisions they can make and why?
- *If not*: Why do you think so?

GBV-related:

1. Could you describe what gender-based violence (change to domestic violence if necessary) means to you?
2. How common do you think that people in your area experience violence within households? 9a. Can you explain why?
3. Could you describe what type of violence [*for prompts: physical, psychological, sexual)* is common? Can you explain why it is more common?
4. Who do you think are the victims? 11a. Can you explain why?
5. Who do you think are the perpetrators? 12a. Can you explain why?
6. What do you think is/are the root cause(s) of this violence in your area? 13a. Can you explain why?
7. If you heard that gender-based violence has taken place, what would you do?
8. Do you think GBV is preventable? 15a. Could you explain why?
9. Is there anything else you would like to tell me about gender-based violence in Tajikistan?

Вопросы на собеседовании

Продолжительность интервью: 60-75 минут

1. Не могли бы вы рассказать о себе?

2. Как долго вы живете в этом сообществе?

Связанные с расширением прав и возможностей:

3. Считаете ли вы, что женщины в вашем сообществе наделены полномочиями или лишены их? 3а. Не могли бы вы объяснить, почему вы так думаете?

⇨ Если ответ таков, что женщины в сообществе наделены полномочиями, спросите:

4. Есть ли в обществе женщины, лишенные прав? 4а. Не могли бы вы объяснить, почему вы так думаете?

5. Как вы считаете, бесправная женщина является жертвой насилия? 5а. Вы можете объяснить почему?

6. Не могли бы вы рассказать мне, как лишенная прав и возможностей женщина может обрести силу в вашем селе / ​​районе?

7. Принимают ли женщины / участвуют в принятии решений в домохозяйствах вашего сообщества?

Для дополнительного зондирования:

⇨ Если да: Можете ли вы описать, какие решения они могут принимать и почему?

⇨ Если нет: почему вы так думаете?

Связанные с гендерным насилием:

8. Не могли бы вы описать, что для вас означает гендерное насилие (переход на домашнее насилие)?

9. Как вы думаете, насколько часто люди в вашем районе сталкиваются с насилием в семье? 9а. Вы можете объяснить почему?

10. Не могли бы вы описать, какой тип насилия (по подсказкам: физический, психологический, сексуальный) распространен? Вы можете объяснить, почему это встречается чаще?

11. Как вы думаете, кто жертвы? 11а. Вы можете объяснить почему?

12. Как вы думаете, кто виновен? 12а. Вы можете объяснить почему?

13. Как вы думаете, что является / являются первопричиной (ами) этого насилия в вашем районе? 13а. Вы можете объяснить почему?

14. Если бы вы узнали о гендерном насилии, что бы вы сделали?

15. Считаете ли вы, что гендерное насилие можно предотвратить? 15а. Не могли бы вы объяснить почему?

16. Что еще вы хотели бы рассказать мне о гендерном насилии в Таджикистане?

Саволҳои мусоҳиба

Давомнокии мусоҳиба: 60-75 дақиқа

1. Оё шумо дар бораи худ нақл карда метавонед?

2. Шумо чанд сол боз дар ин ҷамоат зиндагӣ мекунед?

Вобаста ба ваколатҳо:

3. Оё шумо фикр мекунед, ки занони ҷомеаи шумо қудратманданд ё нотавонанд? 3а. Метавонед шарҳ диҳед, ки чаро шумо чунин фикр мекунед?

⇨ Агар ҷавоб чунин бошад, ки занон дар ҷомеа тавонманданд, лутфан пурсед:

4. Оё дар ҷомеа ягон зане ҳаст, ки маъюб набошад? 4а. Метавонед шарҳ диҳед, ки чаро шумо чунин фикр мекунед?

5. Ба фикри шумо, зани маъюб қурбонии хушунат аст? 5а. Метавонед шарҳ диҳед, ки чаро?

6. Метавонед ба ман бигӯед, ки чӣ гуна як зани маъюб метавонад дар деҳа / ноҳияи шумо қудрат пайдо кунад?

7. Оё занон дар қабули қарорҳо дар хонаводаҳои ҷамоаи шумо / иштирок мекунанд?

Барои санҷиши иловагӣ:

⇨ Агар ҳа: Оё шумо метавонед намуди қарорҳои онҳоро тавсиф кунед ва чаро?

⇨ Агар не: Чаро шумо чунин мешуморед?

Марбут ба GBV:

8. Оё шумо тасвир карда метавонед, ки зӯроварии бар асоси гендерӣ (тағирёбӣ ба хушунати хонаводагӣ) барои шумо чӣ маъно дорад?

9. Ба фикри шумо, то чӣ андоза маъмул аст, ки одамони минтақаи шумо хушунатро дар хонаводаҳо аз сар мегузаронанд? 9а. Метавонед шарҳ диҳед, ки чаро?

10. Оё шумо тасвир карда метавонед, ки кадом намуди зӯроварӣ [барои огоҳкунӣ: ҷисмонӣ, равонӣ, ҷинсӣ] маъмул аст? Метавонед шарҳ диҳед, ки чаро ин бештар маъмул аст?

11. Ба фикри шумо, қурбониҳо кистанд? 11а. Метавонед шарҳ диҳед, ки чаро?

12. Ба фикри шумо, ҷинояткорон кистанд? 12а. Метавонед шарҳ диҳед, ки чаро?

13. Ба фикри шумо, сабаби аслии ин хушунат дар минтақаи шумо чист / аст? 13а. Метавонед шарҳ диҳед, ки чаро?

14. Агар шумо шунидед, ки зӯроварии ҷинсӣ асос ёфтааст, шумо чӣ кор мекардед?

15. Ба фикри шумо, GBV-ро пешгирӣ кардан мумкин аст? 15а. Метавонед шарҳ диҳед, ки чаро?

16. Оё чизи дигаре ҳастед, ки мехоҳед ба ман дар бораи хушунати ҷинсӣ дар Тоҷикистон нақл кунед?

Suhbatga oid savollar

Suhbat davomiyligi: 60-75 daqiqa

1. O'zingiz haqingizda gapirib bera olasizmi?

2. Siz ushbu jamoada qancha vaqt yashadingiz?

Imkoniyatlar bilan bog'liq:

3. Sizningcha, sizning mahallangizdagi ayollar vakolatli yoki kuchsiz deb o'ylaysizmi? 3a. Nima uchun bunday deb o'ylaysiz, tushuntirib bera olasizmi?

⇨ Agar javob jamiyatdagi ayollar vakolatiga ega bo'lsa, iltimos:

4. Jamiyatda imkoniyati cheklangan ayollar bormi? 4a. Nima uchun bunday deb o'ylaysiz, tushuntirib bera olasizmi?

5. Sizningcha, qobiliyatsiz ayol zo'ravonlik qurboni deb o'ylaysizmi? 5a. Buning sababini tushuntirib bera olasizmi?

6. Qanday qilib ishsiz ayol sizning qishloqingizda / tumaningizda kuchga ega bo'lishi mumkinligini ayta olasizmi?

7. Ayollar sizning oilangizdagi uy xo'jaliklarida qaror qabul qilishda / ishtirok etadimi?

Qo'shimcha tekshirish uchun:

⇨ Agar ha bo'lsa: ular qanday qarorlar qabul qilishlarini va nima uchun tasvirlab bera olasizmi?

⇨ Agar yo'q bo'lsa: Nima uchun shunday deb o'ylaysiz?

GBV bilan bog'liq:

8. Jinsiy zo'ravonlik (agar kerak bo'lsa, oiladagi zo'ravonlikka o'zgartirish) siz uchun nimani anglatishini tasvirlab bera olasizmi?

9. Sizningcha, sizning hududingizdagi odamlar uy sharoitida zo'ravonlikka duch kelishadi? 9a. Buning sababini tushuntirib bera olasizmi?

10. Zo'ravonlikning qaysi turi (jismoniy, psixologik, jinsiy) tez-tez uchraydiganligini aytib bera olasizmi? Nima uchun bu ko'proq tarqalganligini tushuntirib bera olasizmi?

11. Sizningcha qurbonlar kimlar? 11a. Buning sababini tushuntirib bera olasizmi?

12. Jinoyatchilar kimlar deb o'ylaysiz? 12a. Buning sababini tushuntirib bera olasizmi?

13. Sizning hududingizda ushbu zo'ravonlikning asosiy sababi (sabablari) nima deb o'ylaysiz? 13a. Buning sababini tushuntirib bera olasizmi?

14. Agar siz jinsga asoslangan zo'ravonlik sodir bo'lganligini eshitgan bo'lsangiz, nima qilgan bo'lar edingiz?

15. Sizningcha, GBV ning oldini olish mumkinmi? 15a. Buning sababini tushuntirib bera olasizmi?

16. Tojikistondagi gender zo'ravonligi haqida menga yana bir narsa aytib berishni xohlaysizmi?
