# Supplementary material for: Exploring the differences between men’s and women’s perceptions of gender-based violence in rural Tajikistan: a qualitative study
Source: BMC Womens Health. 2021 Mar 4;21:91. doi: 10.1186/s12905-021-01227-2 (PMC7934274; doi:10.1186/s12905-021-01227-2)
Supplement: Supplementary file 3 — Additional file 3. Interview prompt for community workshops. [file 12905_2021_1227_MOESM3_ESM.docx]

**Checklist for Workshop**

| ☐ | Date: ___________________________  Name of Facilitator: ______________________________ |
| --- | --- |
| ☐ | What group? ☐ women          ☐ men |
| ☐ | Let everyone know that you will be recording the session, and it will remain anonymous and private. |
| ☐ | Turn on the recorder on the tablet. |
| ☐ | Please include the following:  District name: ______________________    Village name: _________________________ |
| ☐ | Please include the following:  Average age:  ☐ 18 – 25          ☐ 26 – 35       ☐ 36 – 45      ☐ 45+ |
| ☐ | Please include the following:  Average ethnicity:  ☐ Tajik           ☐ Uzbek         ☐ Other (please write): ________________________ |
| ☐ | Notes: |

**СПИСОК ДЛЯ ОТМЕТОК – ДЛЯ СЕМИНАРА**

| ☐ | Дата: _________________________________  Имя Фасилитатора: ___________________________________ |
| --- | --- |
| ☐ | Какая группа ☐ женская или ☐ мужская |
| ☐ | Проинформируйте участников семинара, что вы будете записывать обсуждения на планшет. Дайте им знать что все собранные данные не будут ни с кем делиться и что вся информация останется анонимной. |
| ☐ | До начала семинара, включите планшет и оставьте его записывать обсуждения семинара. |
| ☐ | Пожалуйста запишите:  Название района: ___________________________________________  Название кишлака: __________________________________________ |
| ☐ | Сколько участников: _________________ |
| ☐ | Пожалуйста отметьте средний возраст большинства участников:  ☐ 18 – 25 ☐ 26 – 35 ☐ 36 – 45 ☐ 45+ |
| ☐ | Пожалуйста отметьте среднюю национальность большинства участников:  ☐ Таджики ☐ Узбеки  ☐ Другие (укажите какая): _____________________ |
|  | Для примечаний: |
